# Cost-effectiveness of Pulmonary Rehabilitation Among US Adults With Chronic Obstructive Pulmonary Disease

**DOI:** 10.1001/jamanetworkopen.2022.18189

**Published:** 2022-06-22

**Authors:** Christopher L. Mosher, Michael G. Nanna, Oliver K. Jawitz, Vignesh Raman, Norma E. Farrow, Samia Aleem, Richard Casaburi, Neil R. MacIntyre, Scott M. Palmer, Evan R. Myers

**Affiliations:** 1 Division of Pulmonary, Allergy, and Critical Care Medicine, Duke University Medical Center, Durham, North Carolina; 2 Duke Clinical Research Institute, Durham, North Carolina; 3 Section of Cardiovascular Medicine, Yale School of Medicine, New Haven, Connecticut; 4 Department of Surgery, Duke University Medical Center, Durham, North Carolina; 5 Department of Pediatrics, Duke University School of Medicine, Durham, North Carolina; 6 Rehabilitation Clinical Trials Center, Lundquist Institute for Biomedical Innovation at Harbor-UCLA Medical Center, Torrance, California; 7 Division of Women’s Community and Population Health, Department of Obstetrics and Gynecology, Duke University Medical Center, Durham, North Carolina

## Abstract

**Importance:**

Pulmonary rehabilitation (PR) after exacerbation of chronic obstructive pulmonary disease (COPD) is effective in reducing COPD hospitalizations and mortality while improving health-related quality of life, yet use of PR remains low. Estimates of the cost-effectiveness of PR in this setting could inform policies to improve uptake.

**Objective:**

To estimate the cost-effectiveness of participation in PR after hospitalization for COPD.

**Design, Setting, and Participants:**

This economic evaluation estimated the cost-effectiveness of participation in PR compared with no PR after COPD hospitalization in the US using a societal perspective analysis. A Markov microsimulation model was developed to estimate the cost-effectiveness in the US health care system with a lifetime horizon, 1-year cycle length, and a discounted rate of 3% per year for both costs and outcomes. Data sources included published literature from October 1, 2001, to April 1, 2021, with the primary source being an analysis of Medicare beneficiaries living with COPD between January 1, 2014, and December 31, 2015. The analysis was designed and conducted from October 1, 2019, to December 15, 2021. A base case microsimulation, univariate analyses, and a probabilistic sensitivity analysis were performed.

**Interventions:**

Pulmonary rehabilitation compared with no PR after COPD hospitalization.

**Main Outcomes and Measures:**

Net cost in US dollars, quality-adjusted life-years (QALYs), and incremental cost-effectiveness ratio.

**Results:**

Among the hypothetical cohort with a mean age of 76.9 (age range, 60-92) years and 58.6% women, the base case microsimulation from a societal perspective demonstrated that PR resulted in net cost savings per patient of $5721 (95% prediction interval, $3307-$8388) and improved quality-adjusted life expectancy (QALE) (gain of 0.53 [95% prediction interval, 0.43-0.63] years). The findings of net cost savings and improved QALE with PR did not change in univariate analyses of patient age, the Global Initiative for Obstructive Lung Disease stage, or number of PR sessions. In a probabilistic sensitivity analysis, PR resulted in net cost savings and improved QALE in every one of 1000 samples and was the dominant strategy in 100% of simulations at any willingness-to-pay threshold. In a 1-way sensitivity analysis of total cost, assuming completion of 36 sessions, a single PR session would remain cost saving to $171 per session and had an incremental cost-effectiveness ratio of $884 per session for $50 000/QALY and $1597 per session for $100 000/QALY.

**Conclusions and Relevance:**

In this economic evaluation, PR after COPD hospitalization appeared to result in net cost savings along with improvement in QALE. These findings suggest that stakeholders should identify policies to increase access and adherence to PR for patients with COPD.

## Key Points

**Question:**

Among patients with chronic obstructive pulmonary disease (COPD), is pulmonary rehabilitation (PR) after COPD hospitalization cost-effective in the US health care system?

**Findings:**

In this economic evaluation using data from published literature, a Markov microsimulation model found that PR after COPD hospitalization resulted in net cost savings.

**Meaning:**

These findings provide evidence for stakeholders to use to support polices that will increase access and adherence to PR for patients with COPD.

## Teaser

This economic evaluation compares the estimated effects of posthospitalization pulmonary rehabilitation on cost and quality-adjusted life expectancy in US adults with chronic obstructive pulmonary disease (COPD).

## Introduction

Chronic obstructive pulmonary disease (COPD) is estimated to affect 24 million people in the US and is a leading cause of morbidity and mortality of adults in both the US and across the globe.^1,2,3^ There are significant direct health system costs associated with COPD, namely an estimated $800 billion during the next 20 years.^4^ More than 25% of these costs are attributable to hospitalization for acute exacerbation of COPD,^5^ with one-quarter of these patients readmitted within 30 days.^6^

Pulmonary rehabilitation (PR) involves supervised instruction in exercise training, education, and behavioral change designed to improve physical function and change behavior.^7^ Participation in PR has been shown to relieve breathlessness, increase exercise capacity, and improve health-related quality of life in individuals with COPD.^8,9^ Furthermore, PR has been found to be associated with a significant reduction in hospital admissions and 1-year mortality after hospitalization.^8,10^ Despite consistent evidence of benefits in both randomized clinical trials^8^ and large observational studies,^10,11^ uptake of PR remains low.^12^ Lack of access to transport^13^ and copayments^14^ have been cited as major hurdles to uptake and adherence, whereas others have pointed to poor reimbursement as the critical barrier to broader use.^15^

Although prior studies have demonstrated the cost-effectiveness of PR outside the US,^16,17,18,19^ to our knowledge no estimates of the cost-effectiveness of PR in the US health care system have been published.^7^ Evidence supporting the cost-effectiveness of PR within the US health care system would provide a strong justification to motivate the development of policies to improve use of PR. In this study, we compared the estimated effects of posthospitalization PR on cost and quality-adjusted life expectancy (QALE) measured in quality-adjusted life-years (QALYs) in the US.

## Methods

**Figure 1.**
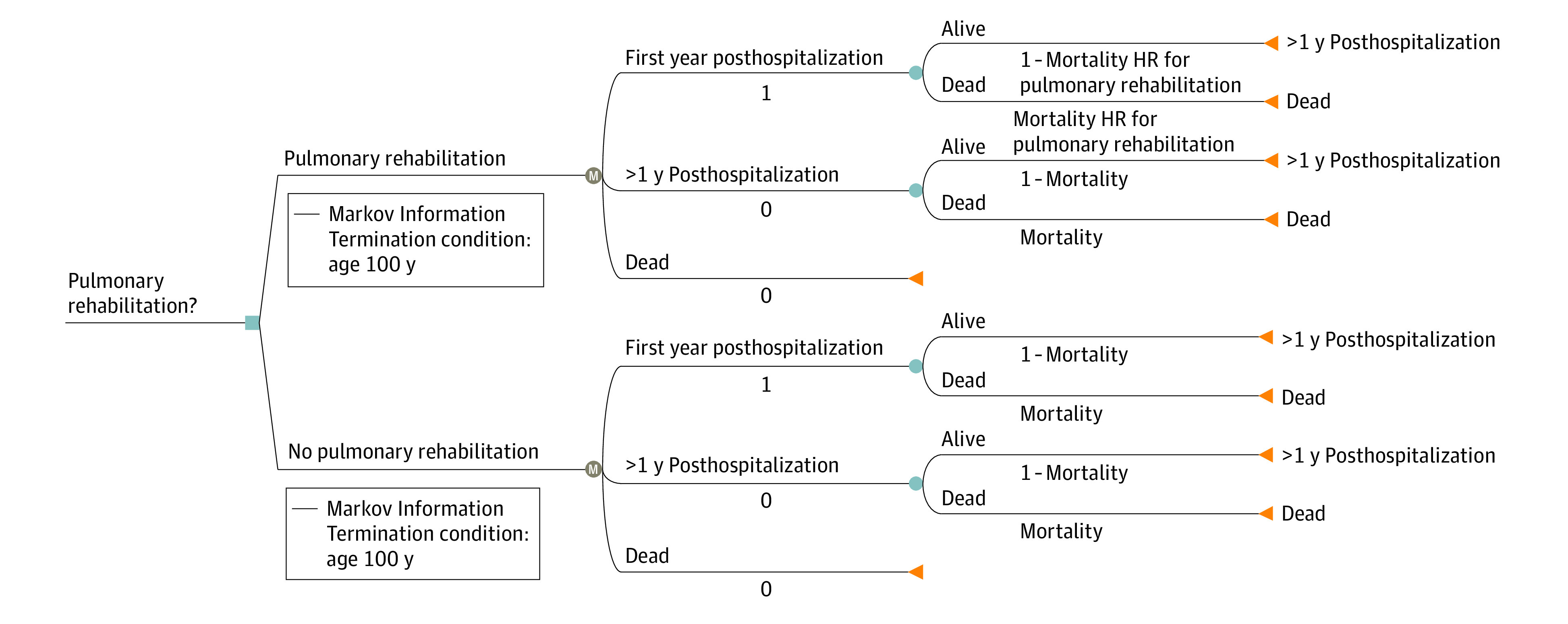
Schematic of Decision Model HR indicates hazard ratio; M, Markov model state.

In this economic evaluation, we constructed a Markov microsimulation model of outcomes after discharge for a COPD hospitalization and compared a strategy of universal PR with no PR in the US health care system (Figure 1). The Markov model itself has 3 states: alive during the first year after the index COPD hospitalization, alive during subsequent years, and dead. Although we used a lifetime time horizon, we assumed that (1) PR would only be performed within 90 days after the index admission; (2) PR would not continue beyond the first year; and (3) PR had no effects, either positive or negative, on cost or outcomes beyond the first year. During the first year, probabilities of readmission or death were conditioned on receipt of PR, as were costs associated with rehospitalization, emergency department (ED) visits, and skilled nursing facility (SNF) stays. Each individual “patient” during the microsimulation was subject to an annual probability of readmission or death. We assumed that PR had no effects after the first year, which meant that subsequent mortality after the first year was dependent only on age, sex, and COPD disease stage defined by the Global Initiative for Obstructive Lung Disease (GOLD) criteria.^20^ Data sources included published literature from October 1, 2001, to April 1, 2021, with the primary source being an analysis of Medicare beneficiaries living with COPD between January 1, 2014, and December 31, 2015 (mean age, 76.9 [range, 60-92] years; 58.6% women). The analysis was designed and conducted from October 1, 2019, to December 15, 2021. Markov cycles were 1 year in length, and costs and outcomes were discounted at a 3% annual rate. The analysis was performed using TreeAge Pro Healthcare, version 2022 (TreeAge Software, LLC). This study followed the Consolidated Health Economic Evaluation Reporting Standards (CHEERS) reporting guideline. All data were publicly available, deidentified data from published studies and trials; therefore, informed consent was not required. Since the study used data from the published literature, the study was exempt from institutional review board review.

### Probability Parameters

Most of the model parameters directly relevant to PR in the US were derived from 2 analyses of fee-for-service Medicare beneficiaries 65 years or older who were hospitalized for COPD in 2014.^10,11^ In these analyses, participants were stratified into those who started PR within 90 days of the index admission and those who either received no PR or started PR more than 90 days after the index admission, with propensity matching used to adjust for baseline differences. We used reported estimates from the propensity-matched cohorts for 1-year mortality,^10^ hospital readmission,^11^ and number of days per person-year in the hospital, ED, or SNF for each group^11^; outcomes reported as rates (such as mortality and readmission) were converted to probabilities using standard methods.^21^ Conservatively, we did not model a dose-response with PR, although Lindenauer et al^10^ reported a statistically significant association between the number of completed sessions and mortality reduction. 1 − Mortality HR for pulmonary rehabilitation

To estimate QALE, we incorporated GOLD stage–specific utilities derived from a previously published large multinational study^22^ that reported EuroQoL-5D scores derived from a US value set stratified by GOLD stage^22^ (Table 1). To account for PR participation population differences in utilities by GOLD stage, we used a published distribution of 30% for GOLD stage 2 (moderate), 48% for GOLD stage 3 (severe), and 22% for GOLD stage 4 (very severe).^23^ To account for the expected improvement in health-related quality of life associated with PR,^8^ we used a reported change in utility.^24^ Although prior studies have shown a negative correlation between utility scores and number of hospitalizations,^25^ we conservatively did not account for this potential additional benefit. To avoid potential underestimation of net lifetime cost attributable to COPD resulting from increased survival after PR, we included life expectancy after the first year posthospitalization using annual all-cause mortality stratified by age, sex, and GOLD stage (Table 1).^20^

**Table 1.** Probability and Utility Parameters

Parameter	Base case	Distribution	Source
Patient characteristics			
Age, mean (range), y			
Main analysis	77 (60-92)	Normal	Stefan et al,^11^ 2021
Sensitivity analysis	65 (50-85)	NA	Assumption
Women (main analysis), % (95% CI)	58.6 (58.4-58.8)	β (α, 115 690; β, 81 686)	Stefan et al,^11^ 2021
COPD stage, %			
GOLD stage 2 (moderate)	30	Dirichlet (α_1_, 75)	Huijsmans et al,^23^ 2008
GOLD stage 3 (severe)	48	Dirichlet (α_2_, 121)	Huijsmans et al,^23^ 2008
GOLD stage 4 (very severe)	22	Dirichlet (α_3_, 57)	Huijsmans et al,^23^ 2008
COPD PR			
No. of sessions			
Fixed (range)	16 (8-36)	NA	Spruit et al,^7^ 2013
Observed, median (IQR)	9 (4-14)	Poisson	Lindenauer et al,^10^ 2020
Hospital readmission, mean (95% CI), d^a^			
Early PR	7.9 (0-63)	γ	Stefan et al,^11^ 2021
Late or no PR	11.7 (2.3-45.3)	γ	Stefan et al,^11^ 2021
No. of ED visits, mean (95% CI)^a^			
Early PR	1.0 (0.2-3.8)	γ	Stefan et al,^11^ 2021
Late or no PR	1.1 (0.2-4.3)	γ	Stefan et al,^11^ 2021
SNF, mean (95% CI), d^a^			
Early PR	1.8 (0-20)	γ	Stefan et al,^11^ 2021
Late or no PR	2.97 (0.5-11.8)	γ	Stefan et al,^11^ 2021
Mortality at 1 y			
Late or no PR, % (95% CI)	14.1 (12.8-15.4)	β (α, 382; β, 2328)	Lindenauer et al,^10^ 2020
Early PR, HR (95% CI)	0.50 (0.42-0.59)	Log normal	Lindenauer et al,^10^ 2020
Mortality rates after first year			
GOLD stage 2, 3 or 4, %			
Men, not varied	2.17-3.97	NA	Shavelle et al,^20^ 2009
Women	1.68-3.07	NA	Shavelle et al,^20^ 2009
Rehospitalization within first year			
Late or no PR, % (95% CI)	63.8 (62.1-65.7)	β (α, 1732; β, 972)	Stefan et al,^11^ 2021
Early PR, HR (95% CI)	0.83 (0.77-0.90)	Log normal	Stefan et al,^11^ 2021
Utilities			
EQ-5D utility score by COPD disease stage, mean (parametric 95% CI)			
GOLD stage 2 (moderate)	0.832 (0.821-0.843)	β	Rutten-van Mölken et al,^22^ 2006
GOLD stage 3 (severe)	0.803 (0.790-0.816)	β	Rutten-van Mölken et al,^22^ 2006
GOLD stage 4 (very severe)	0.731 (0.699-0.762)	β	Rutten-van Mölken et al,^22^ 2006
Utility response to PR, mean (95% CI)	0.065 (0.047-0.083)	Normal	Nolan et at,^24^ 2016

Abbreviations: COPD, chronic obstructive pulmonary disease; ED, emergency department; EQ-5D, EuroQoL-5D; GOLD, Global Initiative for Obstructive Lung Disease; HR, hazard ratio; NA, not applicable; PR, pulmonary rehabilitation; SNF, skilled nursing facility.

^a^ Per person-year.

### Cost Parameters

#### Pulmonary Rehabilitation

All costs were converted to 2020 US dollars using the medical care component of the Consumer Price Index.^26^ We used Centers for Medicare & Medicaid Services reimbursement for COPD PR (billing code G0424) and assumed each PR session would last 2 hours at $44.52/h, and also included patient copayments of $22.28 for each 2-hour PR session (Table 2). For the societal perspective analysis, we added estimates for the costs of travel to and from each session based on previously published mean distance to the PR center.^8,11^ In the base case, we used the distribution of the number of PR sessions observed in the study by Lindenauer et al^10^; as a sensitivity analysis, we varied a fixed rate to 24 sessions to match guidelines of 3 weekly sessions for 8 weeks (Table 1).^7^ Although the comparator groups in both the studies of Lindenauer et al^10^ and Stefan et al^11^ included patients who received some PR starting more than 90 days after the index admission, we did not include any costs associated with these late sessions because only 1.6% of the total no PR group participated in PR after 90 days.

**Table 2.** Costs for COPD PR and COPD-Related Hospital Readmission in 2020 US Dollars

Cost components of COPD PR	Base case	Distribution	Source
2-h session^a^	NA	NA	AACVPR Fact Sheet^27^
Patient copayment, mean, $	22.28	NA	AACVPR Fact Sheet^27^
Travel			
Distance, mean (95% CI), miles	9.9 (0.5-46)	Log-normal	Lindenauer et al,^10^ 2020
Fuel, mean, $ per gallon	2.32	NA	US Bureau of Labor Statistics^26^
COPD-related visits			
Hospitalization per day, $			
Age 45-64 y	2385	NA	HCUPnet^28^
Age 65-84 y	2326	NA	HCUPnet^28^
Age ≥85 y	2338	NA	HCUPnet^28^
ED per nonadmission visit, mean (95% CI), $	922 (228-2725)	γ	Dalal et al,^29^ 2011
SNF per day, median (range), $	255 (173-500)	Uniform	Genworth^30^
Annual per-person COPD-attributable costs after first year by COPD stage, $ (95% CI)			
GOLD stage 2 (moderate)	3858 (694-15 914)	γ	Zafari et al,^4^ 2021
GOLD stage 3 (severe)	5908 (1063-24 371)	γ	Zafari et al,^4^ 2021
GOLD stage 4 (very severe)	6721 (1209-27 724)	γ	Zafari et al,^4^ 2021

Abbreviations: AACVPR, American Association of Cardiovascular and Pulmonary Rehabilitation; COPD, chronic obstructive pulmonary disease; ED, emergency department; GOLD, Global Initiative for Obstructive Lung Disease; HCUPnet, Healthcare Cost and Utilization Project; NA, not applicable; PR, pulmonary rehabilitation; SNF, skilled nursing facility.

^a^ Determined by Centers for Medicare & Medicaid Services code G0424 for PR, including exercise (includes monitoring) 1 hour per session to 2 sessions per day.

We did not include potential time lost from work to attend PR sessions in the societal perspective analysis. First, workforce participation among patients living with COPD is low^31^ because of age and/or symptoms, and productivity losses due to absenteeism^4^ or presenteeism^32^ among those who are employed are common. Although estimating mean wages among employed patients with COPD based on age, sex, and employment sector is theoretically possible, using these data without adjusting for the effects of the disease on productivity would lead to an overestimate of the actual cost. Second, even among patients with COPD who were employed at the time of an index hospitalization, the timing of a postdischarge return to work relative to beginning a PR program is unclear. As a surrogate for potential productivity losses due to either attending PR sessions or postdischarge health care encounters due to exacerbations of disease, we present estimates of the number of days spent undergoing PR and in the hospital, ED, or SNF for each strategy (Table 1). We also did not include potential productivity losses for informal caregivers.

#### Use of Health Care Resources

We estimated the daily cost for a COPD-related hospital admission by identifying hospital discharges in the 2018 Nationwide Inpatient Sample within the diagnostic category of COPD and bronchiectasis^28^ and dividing the mean hospital charges by the mean length of stay (Table 2).^10^ Daily costs for a COPD-related ED visit^29^ were obtained from the literature. For skilled nursing care, we used the median daily cost for a semiprivate room in an SNF reported in the Genworth Survey for 2020 in the base case and varied daily cost using reported state-level medians.^30^ Total cost for each type of admission was estimated by multiplying these daily costs by estimated days. Annual costs after the first year were stratified by GOLD stage based on a recent projection of long-term cost (Table 2).^4^

### Statistical Analysis

In the base case microsimulation, patient-level characteristics (age, sex, and GOLD stage) and outcomes (mortality, utilities, and rehospitalization, ED, and/or SNF days) without PR were drawn from the previously described distributions. The association of PR with outcomes was incorporated by either modifying mortality risk^10^ and utility^24^ based on reported effect estimates or using separate distributions for number of posthospitalization event days (Table 1).^11^

We performed 1-way sensitivity analyses for age, sex, GOLD stage, and number and cost of PR sessions. In addition, we performed scenario analyses assuming (1) no incremental effect of PR on quality of life, (2) no incremental effect on quality of life or mortality, and (3) no incremental effect on quality of life or mortality while varying the hazard ratio for rehospitalization to 1 (ie, no effect). We also performed a 2-way probabilistic sensitivity analysis, using 1000 draws from the effect estimate distributions and 10 000 iterations of the underlying microsimulation.

## Results

In the base case microsimulation from a societal perspective, PR resulted in net cost savings per patient of $5721 (95% prediction interval, $3307-$8388) and an improved QALE (gain of 0.53 [95% prediction interval, 0.43-0.63] years) (Table 3). Most of these savings were owing to reductions in the number of hospital and SNF days (Tables 1 and 3). Savings within the first year after the index hospitalization were $8226 (95% prediction interval, $5348-$10 873); the lower net savings over a lifetime of $5721 are due to higher survival with PR leading to greater longer-term COPD-related costs. From the health system perspective (eliminating patient travel costs), mean savings in the first year were $8667 per patient.

**Table 3.** Estimated Cost and Outcome Intervals

Outcome	Estimate (95% prediction interval)		Difference, PR vs no PR
	No PR strategy	PR strategy	
Net lifetime costs (discounted), $	63 875 (59 187-70 037)	58 154 (53 295-63 335)	−5721 (−3307 to −8388)
Net QALYs	6.89 (6.76-7.03)	7.42 (7.28-7.56)	0.53 (0.43-0.63)
Unadjusted life expectancy, y	8.85 (8.67-9.03)	9.05 (8.86-9.23)	0.20 (0.19-0.20)
Year 1			
Readmission rate, %	63.9 (62.1-65.7)	53.3 (46.5-57.7)	−10.6 (−12.0 to −9.2)
Mortality, %	13.1 (11.9-14.5)	6.3 (5.3-8.0)	−6.8 (−7.6 to −6.0)
QALYs	0.69 (0-0.84)	0.81 (0-0.90)	0.12
Year 1 costs, $			
PR	NA	1749 (1719-1781)	1749
Hospitalization	27 221 (25 822-28 668)	18 360 (17 476-19 247)	−8861 (−8346 to −9421)
ED	1016 (964-1072)	919 (872-968)	−97 (−92 to −104)
SNF	2664 (814-7935)	1647 (498-4851)	−1017 (−316 to −3084)
Net incremental costs, $	NA	NA	−8226 (−5348 to −10 873)
Post–year 1 lifetime costs, $			
Discounted	32 974 (29 194-37 028)	35 479 (31 497-39 963)	2505 (2303-2935)
Undiscounted	33 964 (30 070-38 139)	36 544 (32 442-40 956)	2580 (2372-2817)
After year 1			
QALYs (discounted)	5.4 (0-19.7)	5.8 (0-20.2)	0.4
Undiscounted life expectancy	6.9 (0-25)	7.5 (0-25)	0.6

Abbreviations: ED, emergency department; PR, pulmonary rehabilitation; QALYs, quality-adjusted life-years; SNF, skilled nursing facility.

Use of PR was dominant compared with no PR across sex, age, GOLD stage, and number of sessions (under the assumption of no dose-response with session number) (eTable 1 in the Supplement). If PR does not improve quality of life but only reduces rehospitalizations and mortality, incremental QALYs are 0.41 (vs 0.43). If PR does not improve quality of life or mortality but only prevents readmissions, there are no gains in QALYs, but PR remains cost saving (mean savings of $7607 per patient) unless the hazard ratio for readmission is less than 0.89 (approximately the upper bound of the 95% CI for the observed hazard ratio).

**Figure 2.**
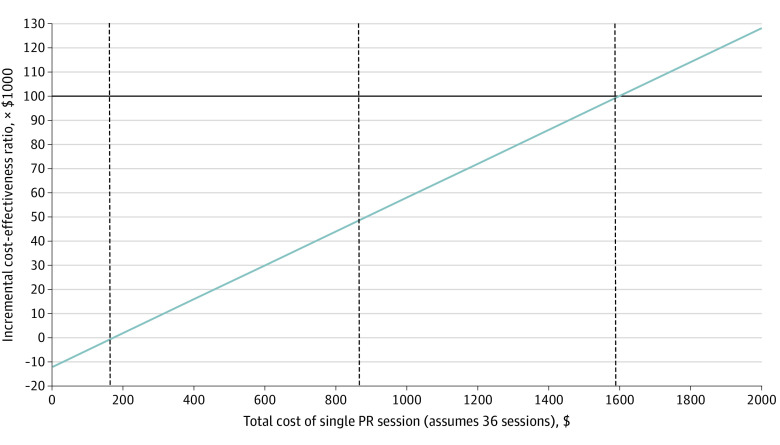
Cost of Single Pulmonary Rehabilitation (PR) Session vs Incremental Cost-effectiveness Ratio At a willingness to pay of $50 000 per quality-adjusted life-year, cost of the PR session was $884; at $100 000 per quality-adjusted life-year, cost of the PR session was $1597.

In probabilistic sensitivity analysis, PR resulted in cost savings and improved QALE and unadjusted life expectancy in every one of 1000 samples of the effectiveness estimates: PR was the dominant strategy in 100% of simulations at any willingness-to-pay threshold (eFigure in the Supplement). As an additional sensitivity analysis, we estimated threshold values for total cost per PR session (2-hour session reimbursement [$89.04] + copay [$22.28] = $111.32/session current state) for a full 36 recommended sessions where PR would no longer be cost saving beginning at $171 per session. Thresholds for the incremental cost-effectiveness ratio of $50 000/QALY and $100 000/QALY were $884 per session and $1597 per session, respectively (Figure 2).

## Discussion

In this model-based analysis, use of PR in the US health care system consistently resulted in net cost savings and improvement in QALE across the range of probability and cost parameters. However, as of 2012, the estimated utilization rate for PR among Medicare beneficiaries was only 4%.^12^ In 2018, there were approximately 393 000 hospitalizations for COPD among Medicare beneficiaries,^28^ with at least one-quarter of these representing readmissions within 30 days after a prior discharge.^6^ Assuming 200 000 patients per year (consistent with the Medicare studies^10,11^) and our estimated savings of $5700 per patient, universal utilization of PR could result in savings for Medicare of $1 to $1.25 billion annually.

We were unable to identify any previously published cost-effectiveness analyses of PR for patients with COPD in the US health care system, although our findings are supported by numerous non-US studies (eTable 2 in the Supplement).^16,17,18,19^ A systematic review by Liu et al^33^ found that PR was cost-effective across a variety of settings, including outpatient and home-based rehabilitation and telerehabilitation.^33^ Although it is reassuring that these prior results are consistent with our present findings, our study has several important differences. In addition to including routinely reported costs of PR, rehospitalization, and ED use,^17,18,19,34^ we included the costs of SNF days, which to date have been reported infrequently (Table 1). In addition, we included data from multiple large data sources using clinical evidence,^10,11^ in contrast to most prior studies that used single-center clinical trial data.^16,17,19^

Although Medicare will cover 1 lifetime PR program (36 sessions) as well as 1 additional program at the request of a physician (total of 72 lifetime sessions),^35^ only 4% of Medicare beneficiaries participate in PR,^12^ compared with 25% of patients with cardiac disease who participate in cardiac rehabilitation.^36^ Low reimbursement has been cited as one of the major barriers contributing toward low PR participation,^15^ and it is worth noting that reimbursement for PR ($44.52) is less than half that for cardiac rehabilitation ($92.84),^15^ despite comparable intensity of service and documentation.^37^ We estimate that the cost of PR per session would remain cost saving until the cost increased to $171 per session for 36 sessions. Furthermore, at a willingness-to-pay of $50 000/QALY, a standard for high-value interventions, PR would remain cost-effective until $884 per session (Figure 2). These findings illustrate the potential cost savings identified in our analysis, which could be used to address reported barriers to patient participation, such as higher reimbursement to support increasing PR building capacity in areas with low density of PR programs,^38^ facilitating patient transportation,^13^ and covering program copayments.^14^

### Limitations

Our study has several limitations. As with any model-based analysis, our results depend on the validity of the model structure; the validity, precision, and applicability of the data used for parameters; and the extent to which all plausible values and scenarios are explored in sensitivity analyses. Most of our estimates of resource utilization come from a large observational study of Medicare beneficiaries that used propensity weighting to account for differences between patients who did and did not use PR.^11^ Major exceptions were for estimates of the distribution of GOLD stages among PR participants^23^ and effect of PR on health-related quality of life,^24^ which came from European studies and may not be generalizable to the US population. We used probabilistic analysis to account for the precision of the estimate of reduction in rehospitalization using Medicare claims data,^11^ which itself was lower than the estimate in a recent Cochrane review based on predominantly Asian and European randomized clinical trials.^8^ In our base case analysis, we used the distribution of attended PR visits (mean of 9) rather than assuming universal completion of a full course of PR. Although this may underestimate cost compared with a full 36 sessions, it may also underestimate effectiveness, because there was a significant correlation between the number of sessions completed and survival.^10^ Whether the estimated cost savings or improvement in health-related quality of life and life expectancy would apply to younger patients, especially if productivity costs were included, is unclear. To the extent that PR reduces posthospitalization use of health care resources among younger patients who may be employed, it seems unlikely that any impact of PR on time away from work would not be mitigated by reductions in ED visits and rehospitalization. We used the most recent Centers for Medicare & Medicaid Services pricing data available to generate national estimates, but because costs vary somewhat by region,^27^ we may have underestimated the cost of PR in specific locations. We did not validate our results because we are not aware of a readily available data set that would allow validation.

## Conclusions

The findings of this economic evaluation suggest that PR after hospitalization for a COPD exacerbation among US patients may result in net cost savings and improvements in QALE. Given these findings, payers—particularly Medicare—should identify policies that would increase access and adherence to PR programs for patients living with COPD.

## Notes

Supplement. **eTable 1.** Effect of Patient Characteristics and Number of Sessions on Costs and Effectiveness **eTable 2.** Summary of Prior Cost-effectiveness Analyses **eFigure.** Scatterplot for Probabilistic Sensitivity Analysis
