# Error in Table

**DOI:** 10.1001/jamanetworkopen.2022.25391

**Published:** 2022-07-14

In the Original Investigation titled, “Cost-effectiveness of Pulmonary Rehabilitation Among US Adults With Chronic Obstructive Pulmonary Disease,”^1^ published June 22, 2022, there was an error in Table 2. The base case cost for a 2-hour pulmonary rehabilitation session should have been listed as $89.04. This article has been corrected.^1^
