# Supplementary material for: Cost-effectiveness of Pulmonary Rehabilitation Among US Adults With Chronic Obstructive Pulmonary Disease
Source: JAMA Netw Open. 2022 Jun 22;5(6):e2218189. doi: 10.1001/jamanetworkopen.2022.18189 (PMC9218844; doi:10.1001/jamanetworkopen.2022.18189)
Supplement: Supplement. — eTable 1. Effect of Patient Characteristics and Number of Sessions on Costs and Effectiveness eTable 2. Summary of Prior Cost-effectiveness Analyses eFigure. Scatterplot for Probabilistic Sensitivity Analysis [file jamanetwopen-e2218189-s001.pdf]

Supplementary Online Content

Mosher CL, Nanna MG, Jawitz OK, et al. Cost-effectiveness of pulmonary rehabilitation among US adults with chronic obstructive pulmonary disease. *JAMA Netw Open*. 2022;5(6):e2218189. doi:10.1001/jamanetworkopen.2022.18189

eTable 1. Effect of Patient Characteristics and Number of Sessions on Costs and Effectiveness

eTable 2. Summary of Prior Cost-effectiveness Analyses

eFigure. Scatterplot for Probabilistic Sensitivity Analysis

This supplementary material has been provided by the authors to give readers additional information about their work.

© 2022 Mosher CL et al. *JAMA Network Open*.

**eTable 1. Effect of Patient Characteristics and Number of Sessions on Costs and Effectiveness**						
**Parameter**	**PR**		**No PR**		**Incremental***	
	**Cost**	**QALY**	**Cost**	**QALY**	**Cost**	**QALY**
**Sex**	NA	NA	NA	NA	NA	NA
**Male**	$54,742	6.48	$60,299	6.01	-$5,557	0.47
**Female**	$59,185	7.46	$64,235	6.92	-$5,050	0.54
**Age**	NA	NA	NA	NA	NA	NA
**65**	$72,266	9.82	$79,115	9.62	-$6,849	0.20
**75**	$54,442	6.18	$61,804	6.06	-$7,362	0.12
**85**	$42,795	4.18	$50,379	4.09	-7,584	0.09
**GOLD Stage**	NA	NA	NA	NA	NA	NA
**2**	$65,775	9.77	$70,949	8.91	-$5,174	0.86
**3**	$54,307	6.64	$58,745	6.19	-$4,438	0.45
**4**	$50,654	4.98	$58,976	4.87	-$8,233	0.11
**Number of PR Sessions**	NA	NA	NA	NA	NA	NA
**10**	$55,835	7.42	$63,875	6.89	-$8,040	0.53
**25**	$57,617	7.42	$63,875	6.89	-$6,258	0.53
**36**	$58,730	7.42	$63,875	6.89	-$5,145	0.53
*PR relative to No PR						

**eTable 2. Summary of Prior Cost-effectiveness Analyses**						
**Outpatient PR program**	**Population**	**Study size**	**Study Design**	**Costs Included**	**Time Horizon**	**Reference**
3 sessions per week for 6 weeks	United Kingdom	200 subjects majority with COPD	RCT	PR program, patient co-pays, outpatient visits and hospitalizations	12 months	Griffiths et al, 16, 2001
2 sessions per week for 4 months	Netherlands	199 GOLD stage 2 or 3 COPD subjects	RCT	PR program, Outpatient visits, hospitalizations, time off work, home care hours, travel expenses	24 months	Hoogendoorn et al, 17, 2010
1 session per week for 8 weeks	Ireland	350 COPD subjects	RCT	PR program, Outpatient visits, hospitalizations, time off work, home care hours,	22 weeks	Gillespie et al, 18, 2013

© 2022 Mosher CL et al. *JAMA Network Open*.

				travel expenses, medications		
2-3 sessions per week for 6-8 weeks	Canada	210 completed PR; majority with COPD, 592 observational controls	Case vs control	PR program, Emergency visits, hospitalizations	12 months	Golmohammadi et al, 19, 2004

eFigure. Scatterplot for Probabilistic Sensitivity Analysis

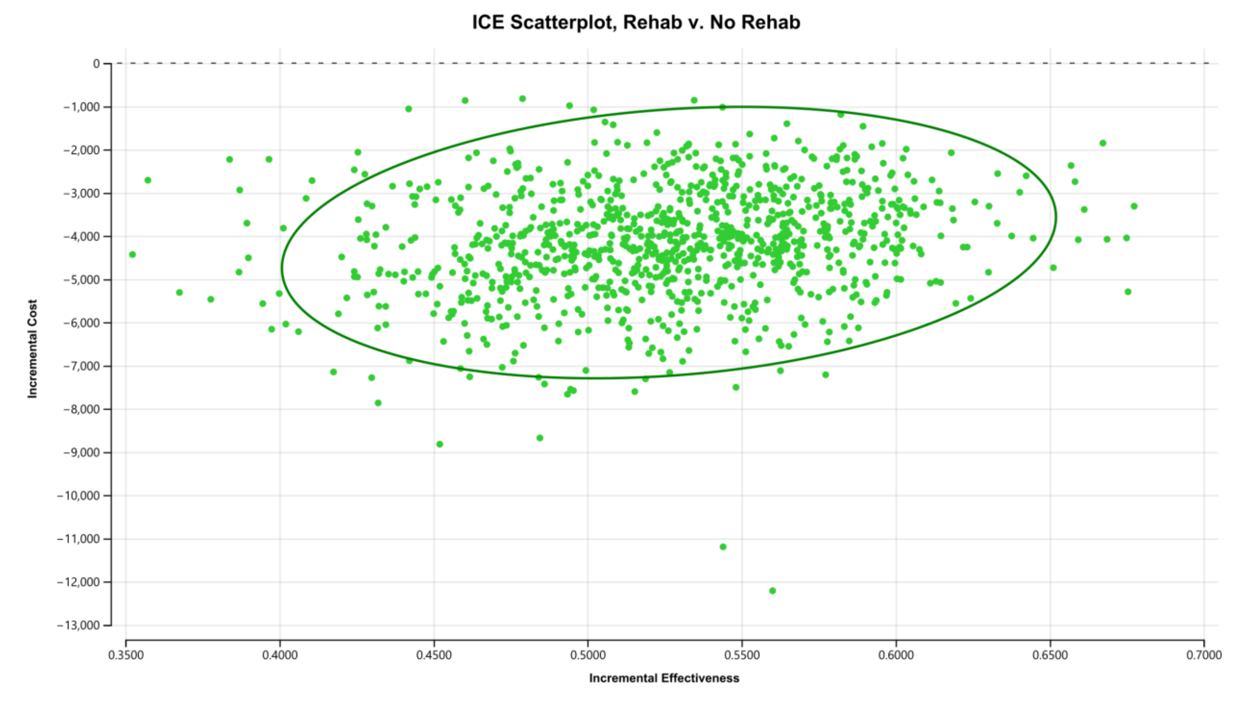

PR resulted in cost-savings and high QALYs in every iteration.

© 2022 Mosher CL et al. *JAMA Network Open*.
